# YY1 Oligomerization Is Regulated by Its OPB Domain and Competes with Its Regulation of Oncoproteins

**DOI:** 10.3390/cancers14071611

**Published:** 2022-03-22

**Authors:** Shiyao Qiao, Wenmeng Wang, Cheng Yi, Qingqing Xu, Wenfei Wang, Jinming Shi, Daniel B. Stovall, Dangdang Li, Guangchao Sui

**Affiliations:** 1 College of Life Science, Northeast Forestry University, Harbin 150040, China; shiyaoqiao@nefu.edu.cn (S.Q.); wangwenmeng@nefu.edu.cn (W.W.); yc6659689@nefu.edu.cn (C.Y.); qqxu@nefu.edu.cn (Q.X.); jmshi@nefu.edu.cn (J.S.); lidd@nefu.edu.cn (D.L.); 2 School of Life Science, Northeast Agriculture University, Harbin 150006, China; wangwenfei@neau.edu.cn; 3 College of Arts and Sciences, Winthrop University, Rock Hill, SC 29733, USA; stovalld@winthrop.edu

**Keywords:** transcription factor, YY1, oligomerization, OPB, zinc finger, oncoprotein

## Abstract

**Simple Summary:**

YY1 regulates various cancer-related genes and activates key oncoproteins. In this study, we discovered that the oncoprotein binding (OPB) domain of YY1 is both necessary and stimulatory to its oligomerization. The hydrophobic residues, especially F219, in the OPB are essential to YY1 intermolecular interaction. Strikingly, the mutations of the hydrophobic residues showed better ability than wild-type YY1 in promote breast cancer cell proliferation and migration. Our further study revealed that YY1 proteins with mutated hydrophobic residues in the OPB domain showed improved binding affinity to EZH2. Overall, our data support the model of a mutually exclusive process between oligomerization of YY1 and its regulation of the oncoproteins EZH2, AKT and MDM2.

**Abstract:**

Yin Yang 1 (YY1) plays an oncogenic role through regulating the expression of various cancer-related genes and activating key oncoproteins. Previous research reported that YY1 protein formed dimers or oligomers without definite biological implications. In this study, we first demonstrated the oncoprotein binding (OPB) and zinc finger (ZF) domains of YY1 as the regions involved in its intermolecular interactions. ZFs are well-known for protein dimerization, so we focused on the OPB domain. After mutating three hydrophobic residues in the OPB to alanines, we discovered that YY1(F219A) and YY1(3A), three residues simultaneously replaced by alanines, were defective of intermolecular interaction. Meanwhile, the OPB peptide could robustly facilitate YY1 protein oligomerization. When expressed in breast cancer cells with concurrent endogenous YY1 knockdown, YY1(F219A) and (3A) mutants showed better capacity than wt in promoting cell proliferation and migration, while their interactions with EZH2, AKT and MDM2 showed differential alterations, especially with improved EZH2 binding affinity. Our study revealed a crucial role of the OPB domain in facilitating YY1 oligomerization and suggested a mutually exclusive regulation between YY1-mediated enhancer formation and its activities in promoting oncoproteins.

## 1. Introduction

As a multiple functional protein, Yin Yang 1 (YY1) is involved in a broad range of biological processes, including cell proliferation, differentiation, cell cycle progression, cellular metabolism, apoptosis, the inflammatory response and oncogenesis [1]. As a master transcription factor, YY1 regulates numerous target genes through binding to its consensus sites in promoters and specific DNA structures, such as G-quadruplexes, and recruiting of P300, CBP and other coactivators to promote gene transcription. YY1 has been reported to activate many cancer-related genes, including MYC, HER2, FOS, SNAIL and TGFβ [1]. Meanwhile, YY1 also shows inhibitory activity in modulating gene expression through its recruitment of corepressors, such as PRC2 and other repressive complexes. Consistently, YY1 has been reported to physically interact with PRC2 components, such as EZH2, and many HDACs [2,3,4,5,6,7]. Among YY1-repressed genes, many of them possess tumor-suppressive activities, including p21, p16INK4A, CEBPD, DR5, microRNA-29 and -206 [1].

Besides the traditional models of transcriptional activation, recent studies provided strong evidence to reveal a pivotal role of YY1 in orchestrating the enhancer–promoter loop formation through its dimerization [8,9,10]. YY1 is present in the active enhancers and promoters of different cell types, and its dimerization can promote enhancer–promoter interactions. As a result, either reduced YY1 expression or its binding element deletion disrupts the enhancer assembly and consequently perturbs gene expression. Therefore, YY1-mediated enhancer–promoter interactions have been considered as a general feature of mammalian gene regulation [8]. YY1 was also demonstrated to form oligomers that could bind to DNA in the absence of its consensus-binding elements [11].

At the posttranslational level, YY1 can also promote oncogenesis in a fashion independent of its transcriptional activity. We and others reported that YY1 destabilizes tumor suppressors p53 and p27 through promoting their ubiquitination and degradation [12,13,14]. Meanwhile, YY1 also binds to the PH domain of AKT to enhance mTORC2-mediated S473 phosphorylation and subsequent activation [15]. Therefore, the general activities of YY1 are proliferative or oncogenic [16]. Consistently, increased YY1 expression has been observed in almost all cancer types compared to their cognate normal cells or tissues [17]. YY1 depletion could block breast cancer cell proliferation and inhibit xenograft tumor formation [14]. In addition, YY1 regulates several hallmarks of cancers, including deregulated cell proliferation, metabolic reprogramming, tumor angiogenesis, cancer cell metastasis and evasion from immune surveillance [18]. All these studies suggested YY1 is a key regulator in the oncogenic process [19]. Recently, based on the OPB sequence and the YY1 binding site on EZH2, we developed two peptides that could disrupt the regulation of oncoproteins by YY1 and consequently reduce the growth of xenograft tumors generated by triple negative breast cancer cells [20,21], which validated YY1 as a cancer therapeutic target.

The fact that multiple oncogenic pathways are regulated by the OPB domain indicates its crucial role in the biological functions of YY1. In the current study, we endeavored to interrogate the contribution of YY1 dimerization or oligomerization to its transcriptional activities. Our results pinpointed the OPB domain as an indispensable region for YY1 oligomerization. Interestingly, mutations of hydrophobic residues, especially F219, could abolish the oligomerization of YY1 but differentially alter its interaction with oncoproteins EZH2, MDM2 and AKT, leading to increased cell proliferation and migration rates.

## 2. Materials and Methods

### 2.1. Plasmids, Peptides and Antibodies

YY1 wild-type (wt) and its mutants 1–226, 1–100, 101–200, 101–226, 154–226, 201–414, 260–414, Δ201–226 (Δ denotes deletion), Δ295–414, Δ201–226, Δ295–414 (i.e., ΔZF, where ZF denotes zinc finger), and the full-length (FL) YY1 mutants with individual amino acid mutations (I212A, L215A, F219A and 3A, with all three residues simultaneously mutated) were generated by PCR amplification followed by subcloning into vectors to express them as GST, His×6, SUMO3 fusion proteins in prokaryotic expression vectors, and/or as HA- and Flag-tagged or EGFP-fusion proteins in mammalian cells, as needed. The oncoprotein binding (OPB) domain of YY1 [15] and its scrambled sequence (WHPQPKKLRCSKSDAAKRRLRGKKIKH) were individually expressed as fusion proteins with mCherry or EGFP fusion proteins in pcDNA3 and/or pSL4 lentiviral vectors. Expression vectors of GST-YY1, His×6-YY1, p53, AKT, MDM2 and EZH2 were reported previously [12,15,22].

To knock down endogenous YY1, a shRNA against human YY1 (shYY1 with a target sequence of GCTCACCTGTTGCTTACAATT at the 3′-UTR of the human YY1 mRNA), and a control shRNA (shCont, with a scrambled target sequence of GGGACTACTCTATTACGTCATT), carried by a lentiviral vector with a puromycin selection marker, were used as we previously reported [23].

The OPB and YPB (YY1 protein binding domain in the human EZH2 protein) peptides, as we previously reported [20], were synthesized by the ChinaPeptides Co., Ltd. (Shanghai, China). The antibodies against GST (cat# PA1-982A from Thermo Fisher Scientific, Waltham, MA, USA), Flag (cat# F3165 from Sigma-Aldrich, St. Louis, MO, USA), mCherry (cat# T0090 from Affinity Biosciences, Cincinnati, OH, USA), His tag (cat# sc-8036, Santa Cruz Biotech., Santa Cruz, CA, USA), YY1 (c-20, SC-218 from Santa Cruz Biotech.), GAPDH (cat# 51332S from Cell Signaling Technology Inc., Danvers, MA, USA) and HA (cat# C29F4 from Cell Signaling Technology Inc.) were used in Western blot analyses.

### 2.2. Cell Culture and Transfection

HeLa cells were cultured in DMEM supplemented with 10% fetal bovine serum (FBS) (Biological Industries USA, Inc., Cromwell, CT, USA) and 1% penicillin/streptomycin (HyClone, Logan, UT, USA). MDA-MB-231 and MDA-MB-453 cells were cultured in RPMI-1640 supplemented with 10% FBS and 1% penicillin/streptomycin. MCF-7 cells were cultured in Alpha-MEM supplemented with 10% FBS, 1% penicillin/streptomycin and 10 mg/mL insulin (Sigma). Lipofectamine 2000 (Invitrogen, Carlsbad, CA, USA) was used in transient transfection using the protocol provided by the manufacturer.

### 2.3. Surface Plasmon Resonance (SPR)

Biacore T200 biosensor (GE Healthsciences) was used in SPR studies. Approximate 12,000 resonance units (RU) of His×6-YY1, His×6-YY1(1–226) and His×6-YY1(260–414) were immobilized individually by amine coupling to the carboxymethylated dextran matrix of the CM5 Chip (GE Healthcare). Purified His×6-p53, SUMO3, wt and mutant YY1 proteins were serially diluted into ladder concentrations of 62.5, 125, 250, 500 and 1000 nM for sample injection. The binding kinetics were analyzed by using Biacore T200 Evaluation software, version 2.0.

### 2.4. Co-Immunoprecipitation (Co-IP) and Western Blot

Cells were lysed in the cell lysis buffer (50 mM Tris-HCl, pH 7.5, 150 mM NaCl, 1% Nonidet P-40, 0.5% sodium deoxycholate and 1% protease inhibitor cocktails) at 4 °C for 15 min, followed by centrifugation at 14,000 rpm, 4 °C for 15 min. Protein concentrations of the supernatants were determined by the Bradford method, and the samples were incubated with anti-Flag beads (cat# B26101, Sigma-Aldrich) at 4 °C for 4 h in a protein binding buffer (50 mM Tris-HCl, pH 7.5, 150 mM NaCl, 1% NP-40, 0.5% sodium deoxycholate and 1% protease inhibitor cocktails). The beads were washed for 7–9 times by the binding buffer and then resuspended in an SDS containing sample buffer. The sample was resolved by SDS-PAGE and then transferred to polyvinylidene difluoride (PVDF) membranes. After being blotted by appropriate antibodies and corresponding secondary antibodies, the immunoreactive bands were visualized using an ECL kit (Vazyme Biotech Co. Ltd., Nanjing, China).

### 2.5. Cell Proliferation Assay

Cell proliferation was determined by the WST-1 assay. Briefly, cells were seeded into a 96-well plate with a density of 3 × 10^3^ cells/well in triplicate and cultured overnight. WST-1 solution (Roche, Indianapolis, IN, USA) was added to each well and incubated at 37 °C for an additional 4 h. Cell proliferation was calculated based on the absorbance at 450 nm, measured by a micro-plate reader (SpectraMax i3) using the GraphPad Prism 5.0 software.

### 2.6. Wound Healing Assay

Cells were cultured in 12-well plates, Scratch wounds were created in each well using a sterile pipette tip, and Mitomycin C was simultaneously added with a final concentration of 1.0 µM. The wounds were imaged at the time points of 0 h (creating the scratches) and 48 h. The migration rates were quantified based on measurement of scratched area at the two time points.

### 2.7. Circular Dichroism (CD) Spectroscopy

Circular dichroism spectra were recorded by a spectropolarimeter (Chirascan; Applied Photophysics Ltd., Surrey, UK) using a quartz cell with a 1.0 mm optical path length. Purified proteins diluted into the concentration of 0.05 mg/mL diluted in PBS were scanned between the wavelengths from 190 to 280 nm at 20 °C. The GraphPad Prism 5.0 software was used to calculate the molar ellipticity data from three individual scans and draw the molar ellipticity curves versus wavelengths.

### 2.8. GST Pull-Down Assay

Recombinant GST fusion proteins expressed and purified from E. coli were lysed with the binding buffer (20 mM Tris·HCl, pH 7.5, 150 mM NaCl, 0.1% Nonidet P-40, 1 mM dithiothreitol, 10% glycerol, 1 mM EDTA, 2.5 mM MgCl_2_ and 1 µg/mL leupeptin) at 4 °C for 30 min. Glutathione Sepharose beads (Thermo Fisher Scientific) were incubated with the bacterial lysates at 4 °C for 4 h. After collecting and washing the beads with cold PBS 6–8 times, 1.0 µg of purified His×6 tagged YY1 was added, followed by additional 4 h of incubation at 4 °C. The washed beads were then resuspended in an SDS-containing sample buffer and analyzed by SDS-PAGE.

### 2.9. Statistical Analysis

The experiments in this study were carried out at least 3 times unless otherwise stated. The software GraphPad Prism 5.0 was used for statistical analysis, and the data are shown as mean ± S.D. Student’s *t*-test and one way ANOVA were employed to assess the statistical significance of differences between data sets. A *p*-value of lower than 0.05 was considered to be significant. *p* < 0.05 (*), *p* < 0.01 (**) and *p* < 0.001 (***).

## 3. Results

### 3.1. The OPB and Zinc Finger Domains Are Involved YY1 Dimerization

**Figure 1:**
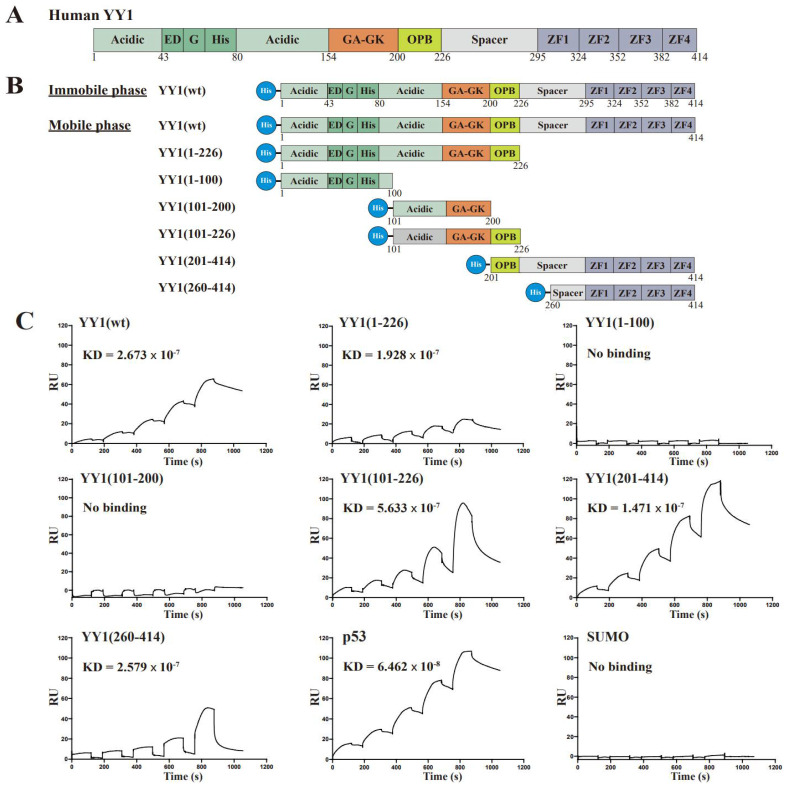

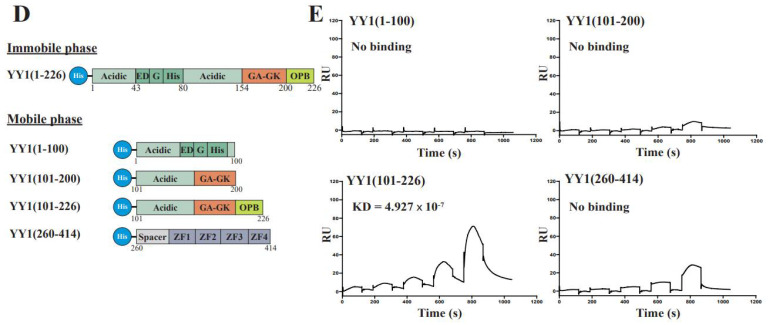
Mapping YY1 binding domains involved in its intermolecular interactions by the surface plasmon resonance (SPR) analysis. (**A**) Schematic diagrams of the domain structures of YY1 protein. “Acidic” represents a region enriched with aspartic and glutamic acids, and “ED” and “G” depict regions containing a glutamic/aspartic acid cluster and a glycine cluster, respectively. “His” indicates a region consisting of 11 consecutive histidines, and “GA” and “GK” represent glycine/alanine- and glycine/lysine-enriched regions, respectively; OPB: oncoprotein binding; the Spacer region and each C2H2-type Zinc finger domain are also denoted. (**B**,**D**) Diagrams of His-tagged (×6) YY1 wt and mutants used to map domains responsible for YY1 intermolecular interactions. (**C**,**E**) Evaluation of YY1 intermolecular interactions using the SPR analysis. As the immobile phase, purified His×6-YY1 wt (**C**) and its mutant (1–226) (**E**) were individually conjugated to the CM5 chip (GE healthcare). As the mobile phase, purified His×6-YY1 wt and its mutant proteins were serially diluted into concentrations of 62.5, 125, 250, 500 and 1000 nM for injection. The samples individually flowed over the chip channels with different conjugates, and the response units (RU) were received from each single cycle. The binding kinetics were analyzed with the Biacore T200 Evaluation software, version 2.0. The results are displayed with time (s) versus and the RU values.

Previous studies suggested that YY1 could form dimers when regulating gene expression [8]. The YY1 protein consists of multiple functional domains (Figure 1A) and has been reported to interact with a number of proteins, but none of them showed detectable binding affinity to the first 154 amino acids of YY1 [1]. To determine the regions responsible for YY1 dimerization, a series of His×6-tagged YY1 truncated mutants was generated (Figure 1B). We employed the surface plasmon resonance (SPR) method to evaluate the interaction of wild-type (wt) YY1 with the YY1 wt and mutants. SPR experiments were carried out using the Biacore T200 biosensor (GE Healthsciences) and the CM5 Chip immobilized with about 12,000 resonance units (RU) of the His×6-YY1(wt) through amine coupling to the carboxymethylated dextran matrix. For the mobile phase, purified His×6-YY1 wt and mutants (Figure 1B) were serially diluted into the concentrations of 62.5, 125, 250, 500 and 1000 nM and individually tested. His×6-YY1(wt) showed decent binding affinity to the immobilized YY1 (with a dissociation constant, KD, of 2.67 × 10^−7^), consistent with its previously reported dimerization ability [8]. We also detected the interaction of YY1 mutants (101–226), (201–414) and (260–414) to His×6-YY1(wt), while the mutants (1–100) and (101–200) did not bind to the immobilized YY1 (Figure 1C). As positive and negative controls, p53 and SUMO exhibited strong binding (KD = 6.46 × 10^−8^) or no binding to YY1, respectively (Figure 1C). Collectively, the data suggested that the two regions involved in YY1 dimerization are located in the 201–226 and 260–414 stretches of the YY1 protein.

We further examined the interactive pattern of YY1 dimerization. After immobilizing the YY1 mutant (1–226) on the CM5 Chip, we found that only YY1(101–226) but not the mutants (1–100), (101–200) and (260–414) could show significant binding (Figure 1D,E). The data indicated that the region of (201–226), the OPB domain responsible for YY1 interaction with multiple oncoproteins [12,15,24], was involved in its dimerization, but it did not interact with the region containing the zinc fingers.

**Figure 2:**
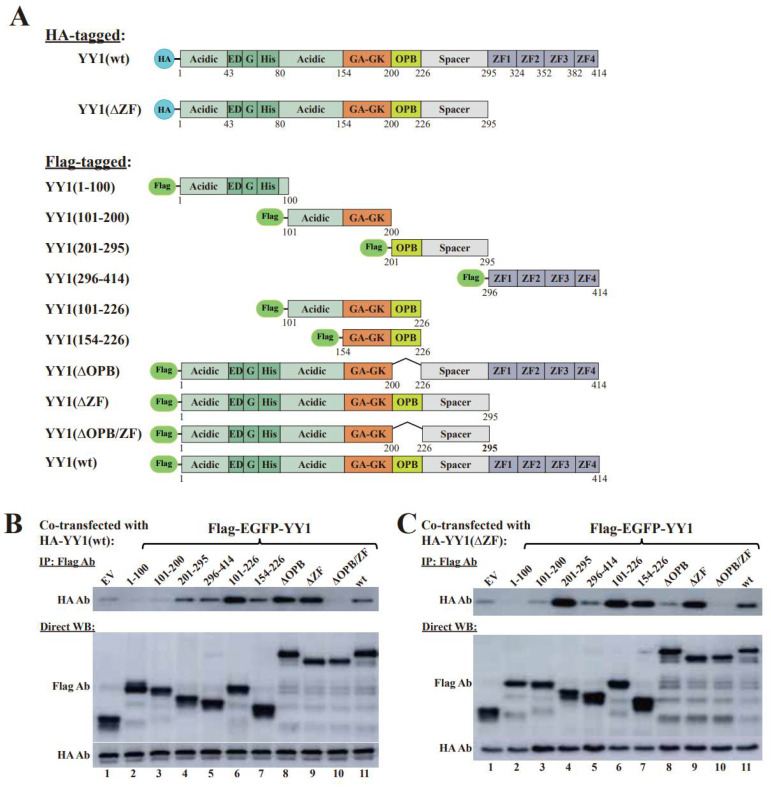

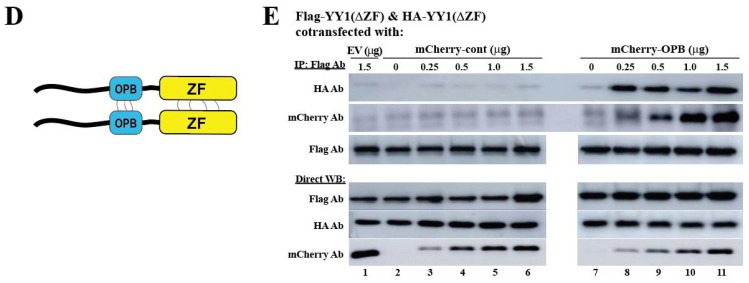
Identification of the binding sites responsible for YY1 dimerization by co-immunoprecipitation (co-IP). (**A**) Diagrams of HA- and Flag-tagged YY1 wt and its mutant proteins used for co-IP studies. (**B**,**C**) Co-IP studies to determine the binding sites responsible for YY1 dimerization. HA-YY1 wt (**B**) or its ΔZF mutant (**C**) expression plasmid was individually cotransfected with Flag-EGFP-YY1 wt and mutant expression vectors, as well as an empty vector (EV). Cell lysates were co-IPed by a Flag antibody, and HA and Flag antibodies were used in Western blot analyses. (**D**) Schematic diagram of a predicted YY1 dimerization mode. (**E**) Effects of the OPB on intermolecular interaction of YY1. Different amounts of mCherry-cont, mCherry-OPB and mCherry plasmids were individually cotransfected with Flag-EGFP-YY1(ΔZF) and HA-YY1(ΔZF) expression vectors, followed by co-IP of the Flag antibody. HA, mCherry and Flag antibodies were used in Western blot analyses.

Due to the insolubility of certain truncated YY1 mutants when purified from bacteria as recombinant proteins, we next carried out co-immunoprecipitation (co-IP) assays to evaluate the interaction of Flag-YY1 wt or its zinc finger (ZF) domain-deleted mutant (ΔZF) with HA-YY1 wt and its mutants (Figure 2A). Consistent with the SPR experiment results, the (1–200) region of YY1 is not involved in YY1 dimerization (lanes 2 and 3 versus 1, Figure 2B,C). Both (201–295) and (295–414, i.e., the ZF domain) were involved in YY1 dimerization (lanes 4 and 5, Figure 2B, and lane 4, Figure 2C), but no significant binding was detected between YY1′s Zinc fingers and its N-terminal-middle region (lane 5, Figure 2C). The immunoprecipitation of the YY1 mutants (101–226) and (154–226) (lanes 6 and 7, Figure 2B,C) suggested the involvement of the OPB domain (i.e., 201–226) in YY1 dimerization. The mutants with individually deleted OPB or ZF domain retained their binding affinity to YY1 wt, but their simultaneous mutations abolished it (lanes 8–10, Figure 2B). However, deletion of both OPB + ZF, or OPB alone, but not ZF domain alone, virtually eliminated the interaction of the generated mutants with HA-YY1(ΔZF) (lanes 8 and 10 versus 9, Figure 2C). Overall, the co-IP results were consistent with the SPR data, indicating that the OPB and ZF domains were responsible for YY1 dimerization, while the two domains did not show cross-interaction (Figure 2D).

Our previous studies indicated the activities of the OPB peptide in blocking YY1 interaction with AKT and EZH2 [15,20], while our data in the current study revealed the involvement of the OPB domain in YY1 dimerization. Therefore, we asked how the OPB peptide could impact YY1 dimerization. For this purpose, we generated two constructs expressing mCherry-OPB and mCherry-cont (a scrambled sequence). To eliminate the effect of the ZF domain, we used the YY1 mutant ΔZF, or (1–295), with Flag- and HA-tags. Co-transfection using Flag-YY1(ΔZF) and HA-YY1(ΔZF), together with increasing amounts of mCherry-cont or mCherry-OPB, was carried out. When IPed by the Flag-antibody, mCherry-OPB, but not the mCherry-cont, could be brought down and markedly enhance Flag-YY1(ΔZF) and HA-YY1(ΔZF) interaction (Figure 2E, original images of all Western blot data are available in File S1), suggesting the role of the OPB domain in promoting YY1 dimerization.

### 3.2. Hydrophobic Residues in the OPB Domain Are Involved in YY1 Dimerization

**Figure 3:**
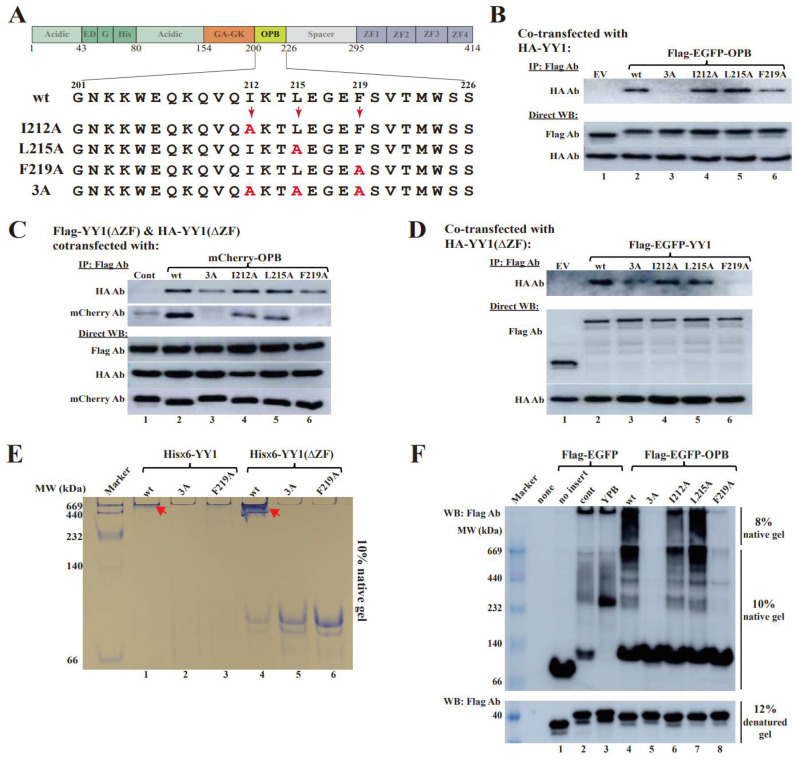

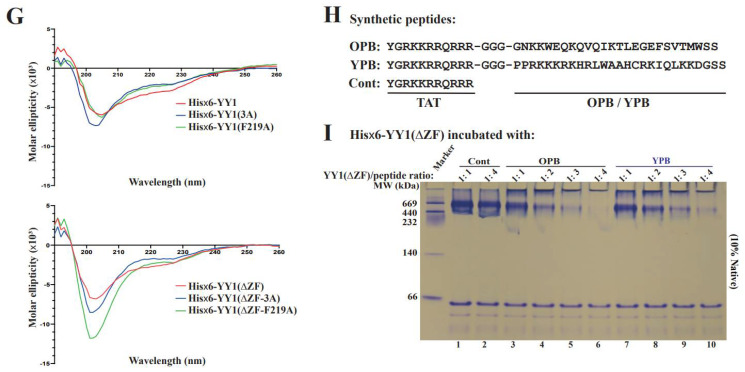
Characterization of key amino acids in charge of YY1 intermolecular interaction. (**A**) Diagrams of OPB point mutations to determine key hydrophobic amino acids involved in intermolecular interactions among YY1 molecules. (**B**) Evaluation of YY1 interaction with OPB wt and mutants. Plasmids of Flag-EGFP-OPB wt and mutants were individually cotransfected with the HA-YY1 expression vector, followed by IP of the Flag antibody and Western blot analyses using labeled antibodies. (**C**) Examination of the effects of OPB wt and mutants on YY1 intermolecular interaction. Plasmids of mCherry-OPB wt, mutants, and cont were individually cotransfected with Flag-YY1(ΔZF) and HA-YY1(ΔZF), followed by IP using the Flag antibody and Western blot analyses using labeled antibodies. (**D**) Evaluation of the interaction between YY1 and YY1 with wt or mutated OPB sequence. Plasmids of Flag-EGFP-YY1(wt) and OPB mutants were individually cotransfected with HA-YY1(ΔZF), followed by IP using the Flag antibody and Western blot analysis using labeled antibodies. (**E**) Native polyacrylamide gel electrophoresis (PAGE) to evaluate the effects of OPB wt and mutants on YY1 intermolecular interactions. Purified His×6-YY1(wt) and (ΔZF) proteins with OPB of wt, 3A or F219A sequences were analyzed by a 10% native PAGE. The red arrow heads denote the oligomerization of YY1 proteins. (**F**) IP studies to examine the binding protein patterns by OPB wt and mutants. HeLa cells were individually transfected by Flag-EGFP-OPB wt, different OPB mutants, YPB, cont and no insert vectors, followed by IP using the Flag antibody, resolved by native gels or SDS-containing gel and analyzed by Western blot using the Flag antibody. (**G**) Circular dichroism spectroscopy of purified YY1 and its mutants. Purified His×6-YY1(wt) and (ΔZF) proteins (0.05 mg/mL) with wt or mutated OPB domain were individually scanned between the wavelengths from 190 to 280 nm at 20 °C. The data were analyzed by the GraphPad Prism 5.0 software. (**H**) The sequences of the synthetic OPB, YPB and Cont peptides. TAT: a cell-penetrating peptide (CPP) derived from human immunodeficiency virus. (**I**) Examination of the effects of OPB and YPB peptides on YY1 oligomerization. Purified His×6-YY1(ΔZF) protein (6.5 µg, or 140 pmol) was mixed with synthetic OPB, YPB and Cont peptides with a serial molar ratio of 1:1, 1:2, 1:3 and 1:4, and incubated at 4 °C for 2 h, followed by the analysis of 10% native PAGE.

Both hydrophobic and electrostatic interactions can promote intermolecular protein association, but hydrophobic binding has been considered as the major driving force for protein oligomerization [25]. For instance, hydrophobic residues of p53 are involved in its protein tetramerization [26]. After scanning the OPB sequence, we identified three major hydrophobic residues, I212, L215 and F219. To evaluate their contribution to YY1 dimerization, we individually or simultaneously mutated them to alanine (Figure 3A). We first tested the binding of these OPB mutants to YY1 by co-transfecting Flag-EGFP-OPB wt or mutants with HA-YY1 vectors, with the cell lysates IPed by the Flag antibody. Compared to the OPB wt, the OPB-3A mutant, with all three hydrophobic residues replaced by alanines, lost the binding affinity to HA-YY1 (Figure 3B, original images of all Western blot data are available in File S1). Meanwhile, among the three single site mutants, OPB(F219A) exhibited markedly reduced ability in bringing down HA-YY1 (Figure 3B).

Next, we examined the effects of these OPB mutants on intermolecular interaction among different YY1 proteins. As shown in Figure 3C, original images of all Western blot data are available in File S1, both F219A and 3A but not I212A and L215A of the mCherry-OPB mutants, showed markedly reduced ability in promoting Flag-YY1(ΔZF) and HA-YY1(ΔZF) interaction. Meanwhile, all OPB mutants, especially F219A and 3A, exhibited decreased binding to Flag-YY1(ΔZF) (Figure 3C).

To evaluate whether the hydrophobic residues in the OPB domain are directly involved in intermolecular interaction of YY1, we created Flag-EGFP-YY1 mutants with altered OPB sequences and determine their interactions with HA-YY1(ΔZF). Compared to the Flag-EGFP-YY1(wt), its mutants (I212A) and (L215A) showed similar binding affinity to HA-YY1(ΔZF), but the (F219A) and 3A mutants virtually lost this ability (Figure 3D, original images of all Western blot data are available in File S1), suggesting that the hydrophobic residues, especially F219, in the OPB domain played an essential role in YY1 dimerization.

We also carried out native polyacrylamide gel electrophoresis (PAGE) to evaluate the interaction among YY1 proteins. The recombinant YY1 with wt, F219A and 3A OPB sequences were individually analyzed by native PAGE on a 10% gel. His×6-YY1(wt) and (ΔZF) with wt OPB showed high molecular weight aggregations at the bottom of the loading wells or right beneath them (Figure 3E), suggesting their ability to form oligomers. For the YY1(F219A) and 3A mutants, His×6-YY1(wt) displayed markedly reduced signal in the loading wells, while their His×6-YY1(ΔZF) proteins mostly formed bands with relatively low molecular weights (Figure 3E), suggesting their lack of ability in forming high molecular weight oligomers. To assess the effects of the wt and mutant OPB in a cellular environment, we transfected pcDNA3-Flag-EGFP-OPB wt and its mutants into HeLa cells (Figure 3F, original images of all Western blot data are available in File S1). Meanwhile, an EGFP-cont (a scrambled sequence), an EGFP-YPB (the YY1 protein binding domain on EZH2, based on our previous study [20]) and a Flag-EGFP vector were also tested. The cell lysates were IPed by the Flag antibody, followed by the analysis on 10% native PAGE. Compared with the vector (no insert) control, the scrambled cont and YPB showed similar bands compared to the control of vector alone (lanes 2 and 3 versus 1). The OPB wt, I212A and L215A could pull down many more, likely similar, components than the cont (lanes 4, 6 and 7 versus 2); however, the OPB 3A and F219A mutants, especially the former one, were incapable of bringing down, or associating with, any component. The data strongly suggest that the hydrophobic residues, especially the F219A, are responsible for the interaction of YY1 with its binding partners, including its oligomerization. Loss of protein activities may be caused by single residue alteration or catastrophic distortion of protein structures. We analyzed purified recombinant His×6-YY1 wt and ΔZF with intact or mutated OPB using circular dichroism (CD) analysis. As shown in Figure 3G, YY1 wt, F219 and 3A exhibited comparable CD spectral patterns in either wt or ΔZF forms, suggesting that the mutations of the hydrophobic residues in the OPB domain did not significantly impact the overall structure of the YY1 protein.

We further tested the effects of the synthetic peptides OPB and YPB (Figure 3H) on the oligomerization of His×6-YY1(ΔZF). In the presence of a control peptide (Cont) at two different concentrations, YY1(ΔZF) showed bands with comparable intensity around 669 kDa (lanes 1 and 2, Figure 3I), suggesting its oligomerization due to the large molecular weight. The presence of increased OPB peptide quickly and monotonically diminished this band and simultaneously generated a stained band in loading wells, likely due to the formation of ultra-large protein complexes (lanes 3 to 6, Figure 3I). However, the band intensity in the loading wells markedly dropped with the increased YY1(ΔZF)/OPB peptide ratio (lanes 5 and 6 versus 3 and 4), possibly due to the washaway of the oversized complexes in loading wells during the gel staining and destaining process. Interestingly, the YPB peptide showed very similar effects to the OPB peptide in promoting the formation of ultra-large YY1(ΔZF) complexes (lanes 7 to 10, Figure 3I), suggesting that any peptide with binding affinity to the OPB domain could robustly promote YY1 super-oligomerization.

### 3.3. Mutations of Hydrophobic Residues in the YY1 OPB Domain Improve Breast Cancer Cell Proliferation and Migration

**Figure 4:**
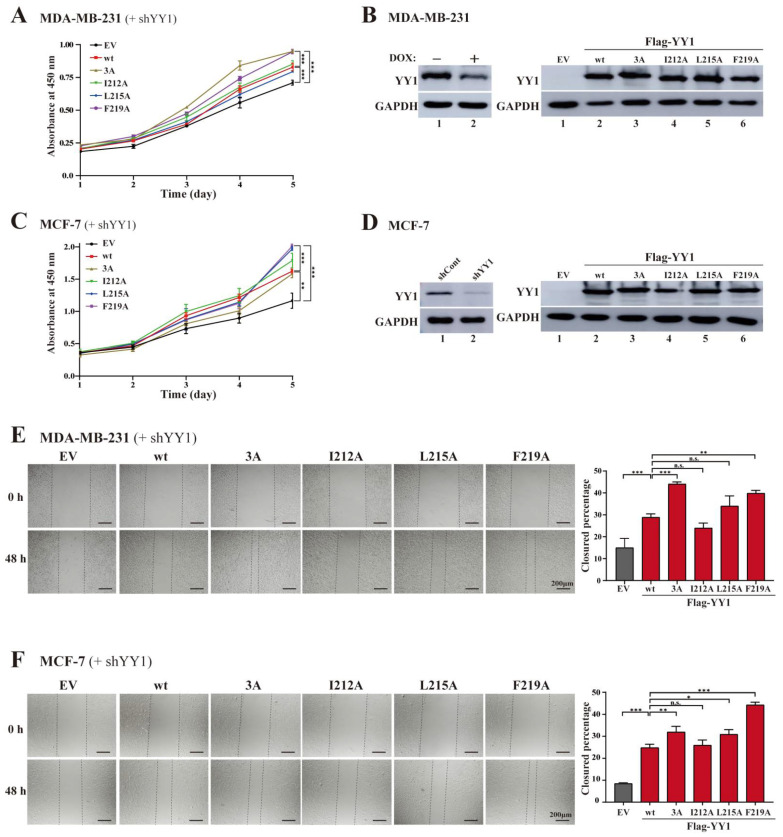
Effects of the OPB mutations on cell proliferation and migration of breast cancer cells. (**A**–**D**) Effects of ectopic expression of Flag-YY1 wt and its OPB mutants on the proliferation of breast cancer cells. MDA-MB-231 cells carrying a TET-inducible shYY1 targeting the YY1 mRNA 3′-UTR cultured in doxycycline (DOX)-containing medium (**A**), or MCF-7 cells infected by lentivirus carrying the same shYY1 (**C**), were infected by lentivirus expressing Flag-YY1 wt and mutants, followed by WST-1 assay, to determine cell proliferation. Endogenous YY1 knockdown and ectopic expression of YY1 wt and mutants in MDA-MB-231 and MCF-7 cells are shown in (**B**,**D**), respectively. (**E**,**F**) Scratch assays to test the effects of YY1 mutants on breast cell migration. Endogenous YY1 knockdown and ectopic YY1 expression in MDA-MB-231 (**E**) and MCF-7 (**F**) cells carried out as described in (**A**–**D**). Images were captured when scratches were just made on the plates of overnight cultured cells and after 48 h of culture in the presence of 1.0 µM of Mitomycin C. The quantitation of cell migration is shown at the right panels. Data represent the mean ± S.D., n.s.: no significance, * *p* < 0.05, ** *p* < 0.01 and *** *p* < 0.001.

To evaluate whether the mutations in the OPB domain could alter the overall biological functions of YY1, we expressed the Flag-YY1 wt and its OPB mutants in MDA-MB-231 and MCF-7 cells with simultaneous knockdown of endogenous YY1 using an shRNA targeting the 3′-UTR of the human YY1 mRNA, as we previously reported [23]. In the MDA-MB-231 cells, ectopic expression of Flag-YY1 wt and its two mutants I212A and L215A showed similar effects in restoring cell proliferation, compared to the empty vector control (Figure 4A,C); however, interestingly, both Flag-YY1(F219A) and (3A) mutants exhibited stronger capacities in promoting cell proliferation than wt YY1 did (Figure 4A). To our surprise, in MCF-7 cells, F219A and L215A mutants could significantly improve cell proliferation, but the 3A and I212A mutants showed comparable activities to that of Flag-YY1 wt (Figure 4C). The discrepancy of the 3A mutant activities in MDA-MB-231 and MCF-7 cells could be due to the different genetic backgrounds between the two cell lines, which needs further investigation. Nevertheless, the results manifested the importance of the hydrophobic residues, especially F219, of the OPB domain in modulating the proliferative activity of YY1. The YY1 shRNA (shYY1) targeting the 3′-UTR of the YY1 mRNA was reported previously [23]. The knockdown efficiency of endogenous YY1, and ectopic expression of Flag-YY1 wt and its mutants, are shown in Figure 4B,D, original images of all Western blot data are available in File S1. In addition, in the scratch assay, MDA-MB-231 and MCF-7 cells expressing the YY1 mutants 3A and I219A also showed faster migration rates than the cells expressing wt YY1 (Figure 4E,F). Meanwhile, MDA-MB-231 cells harboring the YY1(I212A) exhibited slightly reduced migration ability compared to the cells with wt YY1 (Figure 4E). Overall, our functional results of the YY1-OPB mutants suggested that disruption of OPB-mediated dimerization was beneficial to the overall proliferative activity of YY1 in breast cancer cells.

### 3.4. OPB Mutations Differentially Affect YY1′s Interactions with Oncoproteins

**Figure 5:**
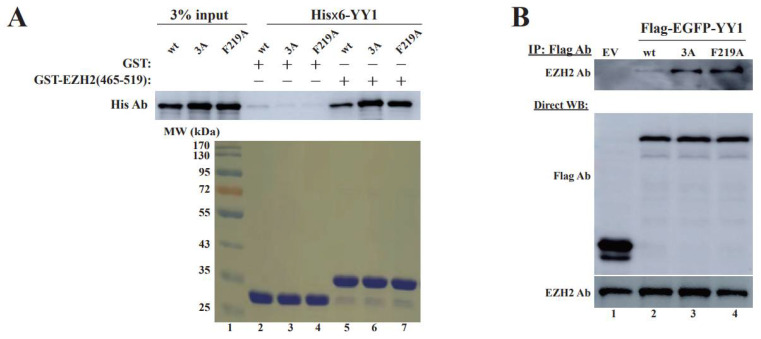

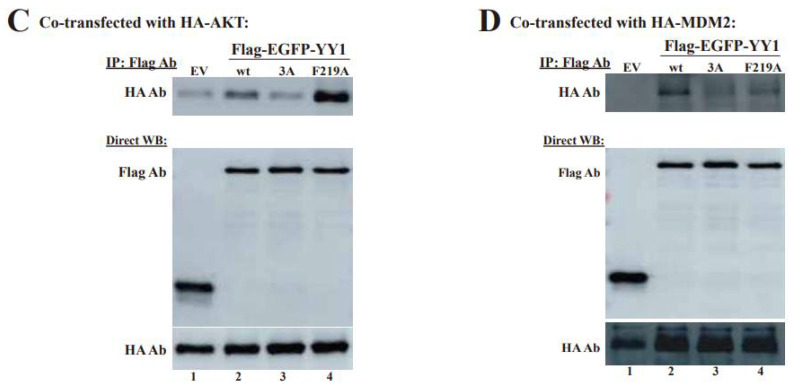
Evaluation of the effects of OPB mutations on YY1 interaction with oncoproteins. (**A**,**B**) Examination of the interaction between YY1 OPB mutants and EZH2. In A, GST and GST-EZH2(465–519) were individually incubated with purified His×6-YY1 wt, 3A and F219A mutants, followed by extensive wash and Western blot analyses using a His-tag antibody. The input of GST and GST-EZH2(465–519) is shown at the lower panel. In (**B**), plasmids of Flag-EGFP-YY1 wt, 3A and F219A were individually transfected into HeLa cells, followed by IP using the Flag antibody and Western blot analyses using an EZH2 antibody. Direct Western blot analyses were conducted using the antibodies as labeled. (**C**,**D**) Examination of the interaction of YY1 OPB mutants with AKT and MDM2. Flag-EGFP-YY1 plasmid was cotransfected with HA-AKT (**C**) or HA-MDM2 (**D**) into HeLa cells, followed by IP using the Flag antibody and Western blot analyses using an HA antibody. Direct Western blot analyses were conducted using the antibodies as labeled. Original images of all Western blot data are available in File S1.

Previous studies indicated that the OPB domain is involved in YY1 binding to EZH2, AKT and MDM2 proteins. Therefore, we tested whether the changes of the three hydrophobic residues could reduce YY1 binding to these oncoproteins. EZH2 is an essential partner of YY1 in promoting cancer cell progression [20,27]. Therefore, we first tested the binding affinity of these YY1-OPB mutants with EZH2. Our previous study mapped the YY1 binding site on EZH2 to the residues 493–519 [20]. We carried out an in vitro protein binding assay using the GST-EZH2(465–519) and observed that its interaction with wt YY1 was less than that with YY1 mutants 3A and F219A (Figure 5A). We also transfected Flag-YY1 vectors into HeLa cells, followed by co-IP using the Flag antibody. As shown in Figure 5B, Flag-YY1 mutants 3A and F219A showed much higher binding affinity to endogenous EZH2 than the wt Flag-YY1 did. In addition, we tested the interaction of the YY1 mutants 3A and F219A with AKT1 and MDM2. Compared to wt YY1, its mutant (F219A) showed increased, while (3A) showed decreased, interaction with HA-AKT (Figure 5C). Additionally, in co-IP studies, both YY1(F219A) and (3A) exhibited lower binding affinity to HA-MDM2 than wt YY1 (Figure 5D). Overall, our data demonstrated that the F219A mutation could abolish the OPB-mediated dimerization of YY1 but differentially alter its interaction with oncoproteins EZH2, AKT1 and MDM2. These observations may provide an explanation for enhanced proliferation of breast cancer cells expressing YY1 mutants F219A and 3A. The reduced YY1 dimerization caused by F219A mutation may subsequently impact the chances of its binding to YY1-regulated oncoproteins and especially improve its interaction with EZH2, to promote their cell proliferative activities. Based on our data and inference, we propose that YY1 dimerization or oligomerization and its interactions with the oncoproteins are competitive or mutually exclusive events. The latter regulations are likely more important to the malignant transformation of cancer cells.

**Figure 6:**
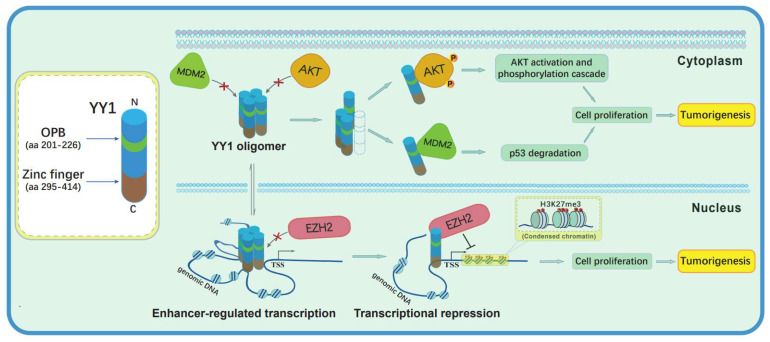
A schematic model of competitive processes between YY1 oligomerization and its interactions with different oncoproteins. In the nucleus, YY1 oligomers may activate target gene expression through facilitating enhancer formation, but may not be able to bind the oncoproteins. As monomers, YY1 interacts with EZH2, AKT and MDM2 in either the nucleus or cytoplasm to promote their oncogenic activities, including suppressing tumor suppressive genes, stimulating proliferative signaling pathways, and enhancing p53 ubiquitination and degradation, respectively.

Based on the results in the current study and previous reports, YY1 may form oligomers that may facilitate enhancer formation to activate gene expression. However, the oligomerization can prevent YY1 from binding and regulating oncoproteins EZH2 in the nucleus and AKT and MDM2 in cytoplasm (Figure 6). Disassociation of the oligomerization may allow EZH2 recruitment by YY1 to its target promoters and subsequent gene repression. Meanwhile, YY1 monomers also interact with AKT to promote its phosphorylation and activation [28], while YY1 interaction with MDM2 enhances p53 ubiquitination and degradation [12]. The regulation of AKT activation and p53 ubiquitination may occur in both the cytoplasm and nucleus [29,30]. In addition, YY1 can also stay in both the nucleus and cytoplasm, especially in cancer cells [14,31].

## 4. Discussion

The regulation of YY1 in different oncogenic signaling pathways depends on its transcriptional activities and direct binding to many oncogene products. In both scenarios, the interactions of YY1 with either transcription cofactors or oncoproteins play a crucial role. In the current study, we focused on dissecting the contribution of YY1 dimerization to its biological activities and observed that YY1 could oligomerize as an in vitro purified recombinant protein. Importantly, we discovered a novel function of the OPB domain in mediating YY1 oligomerization and revealed a determinant role of hydrophobic residues, especially F219, in this domain. Unexpectedly, breast cancer cells harboring YY1 mutants with these hydrophobic residues replaced by alanines showed improved cell proliferation and migration. When interrogating the underlying mechanism of this phenomenon, we tested the interaction of these YY1 mutants with oncoproteins and observed their differentially altered binding affinity to EZH2, AKT and MDM2, which likely contributed to the improved capability of the YY1 mutants in promoting cell malignancy.

During our investigation of the domains involved in YY1 dimerization, we first observed that YY1 formed oligomers, instead of dimers, based on the molecular weights of the smeared bands in native gel; meanwhile, two regions, including the OPB and ZF domains, are involved in the intermolecular interactions of the YY1 protein. Our data also supported the interaction pattern of OPB-OPB and ZF-ZF but not OPB-TF. ZFs are stable molecular scaffolds with sticky properties and may act as protein-recognition motifs [32]. Especially, as the most prevalent protein motif among the proteins produced in mammalian cells, the C2H2 ZF is considered as a bona fide dimerization domain [33]. Therefore, it is not surprising that the ZF domain of YY1, as a C2H2 transcription factor [34], showed activity in mediating YY1 dimerization. Importantly, when the ZF domain of each YY1 protein binds to its consensus sites in genomic DNA, it will leave the OPB domain as the only region for YY1 dimerization or oligomerization, which may contribute to the formation of typical enhancers or even super-enhancers to promote gene expression. Interestingly, a previous study indicated that oligomerized YY1 proteins could bind to chromatin DNA lacking its recognizable consensus elements [11]. Whether YY1 oligomerization may affect the ZF domain structure to alter its flexibility or selectivity during DNA binding remains elusive. Therefore, whether nuclear YY1 forms dimers or oligomers determines its regulatory modes. When staying as monomers but binding to transcriptional cofactors, such as EZH2, YY1 regulates gene expression through a typical or general transcription mechanism. When forming OPB-mediated dimers or oligomers, YY1 may facilitate the connection among distant genomic DNA elements to promote the formation of enhancers or super-enhancers.

In the current study, we initially wanted to delineate molecular mechanism of YY1 dimerization, but our data supported multi-molecular YY1 protein oligomerization. Interestingly, the OPB domain, previously identified as an oncoprotein binding site [15], is required for YY1 intermolecular interaction, and the presence of the OPB peptide, either in a fusion protein or as a synthetic molecule, could greatly facilitate YY1 oligomerization. Currently, the mechanism underlying OPB peptide-promoted formation of YY1 super-complexes is still unclear, but interaction of the OPB peptide with the YY1 OPB domain is likely crucial for this regulation, because the YPB peptide, which also binds the OPB domain [20], also showed similar activity to OPB peptide, and OPB(F219A) and (3A) did not (Figure 3F). Our previous design of OPB and YPB peptides to exert their anticancer activities was based on the assumption that the two peptides could block YY1 interactions with oncoproteins EZH2, MDM2 and AKT [20,21]. However, according to the results of this study, the OPB and YPB peptides may also cause aberrant oligomerization of YY1, which is generally overexpressed in cancer cells, leading to apoptotic cell death.

We have previously demonstrated that lysines, arginines, serines and threonines in the OPB domain are involved in YY1 interaction with AKT [21]. In the current study, mutations of the hydrophobic amino acids in the OPB domain decreased YY1 binding to MDM2 but markedly increased its interaction with EZH2; YY1(F219A) mutant showed improved binding to AKT, but YY1(3A) exhibited reduced AKT interaction (Figure 5). Based on these data, we conclude that different residues are responsible for YY1 intermolecular interaction and its binding to oncoproteins. The hydrophobic amino acids in this region are involved in YY1 dimerization or oligomerization, which are very likely crucial to the transcriptional regulation of YY1 through forming enhancers. These residues are also important for YY1 interaction with MDM2 to maintain low p53 levels. Nevertheless, due to the relatively short length (26 amino acids) of the OPB, the OPB domain-regulated YY1 oligomerization and oncogene activation are very likely mutually exclusive. In the current study, the YY1(F219A) and (3A) mutants showed significantly improved binding to EZH2, with simultaneously increased breast cancer cell proliferation and migration, compared to wt YY1. The data strongly suggested that the YY1-regulated EZH2 activity plays a dominant role in promoting cancer progression. On the other hand, AKT activation and p53 attenuation may have already been well-achieved and maintained in breast cancer cells, which mostly occur in cytoplasm, and the regulation of YY1 in promoting AKT activation and MDM2-mediated p53 degradation may not be prominently required at this stage. Importantly, the staining of antibodies specific to phosphorylated AKT-S473 and -T308, markers of AKT activation, could generate signals predominantly in nuclei [15], and MDM2-mediated p53 ubiquitination in the nucleus has also been frequently reported [30,35,36]. Therefore, the nuclear YY1 statuses as monomers or oligomers in cancer cells not only decide its transcriptional regulatory modes but also modulates its actions to promote AKT1 activation and p53 degradation. As cytoplasmic YY1 was also previously reported [14,37,38,39], the regulation of AKT and p53 by YY1 monomers may certainly occur in cytoplasm. In addition, YY1 dimerization or oligomerization may be important for cell differentiation and embryonic development but indispensable for the proliferation of cancer cells, which are already in a chaotic state of gene expression.

## 5. Conclusions

In this study, we discovered a novel role of YY1′s OPB domain, which was previously identified to directly interact with and regulate multiple oncoproteins, such as AKT, MDM2 and EZH2. We revealed that the hydrophobic residues, especially F219, in the OPB, are essential to YY1 oligomerization. Furthermore, we uncovered the counteractive regulation between YY1-mediated enhancer formation and oncogenic activation.

## Data Availability

The data presented in this study are available in this article (and Supplementary Material).

## Supplementary Materials

The following supporting information can be downloaded at: https://www.mdpi.com/article/10.3390/cancers14071611/s1, File S1: The supplementary materials contain original experimental images.
