# Supplementary material for: YY1 Oligomerization Is Regulated by Its OPB Domain and Competes with Its Regulation of Oncoproteins
Source: Cancers (Basel). 2022 Mar 22;14(7):1611. doi: 10.3390/cancers14071611 (PMC8996997; doi:10.3390/cancers14071611)

cancers

Original Figure
Figure 2B:
IP; HA Ab

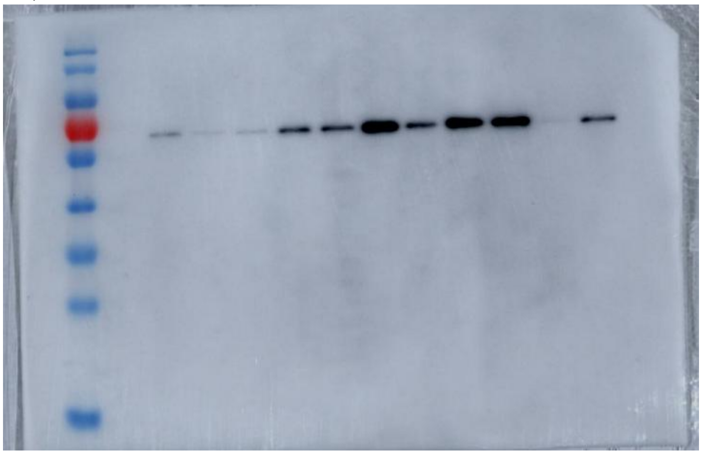

Direct WB; Flag Ab

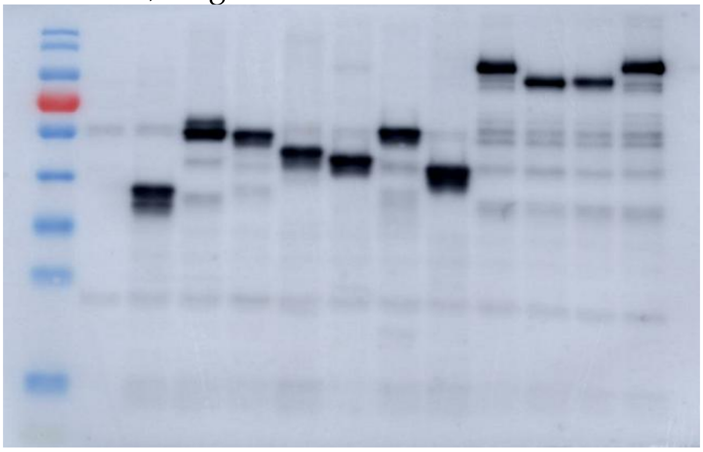

Direct WB; HA Ab

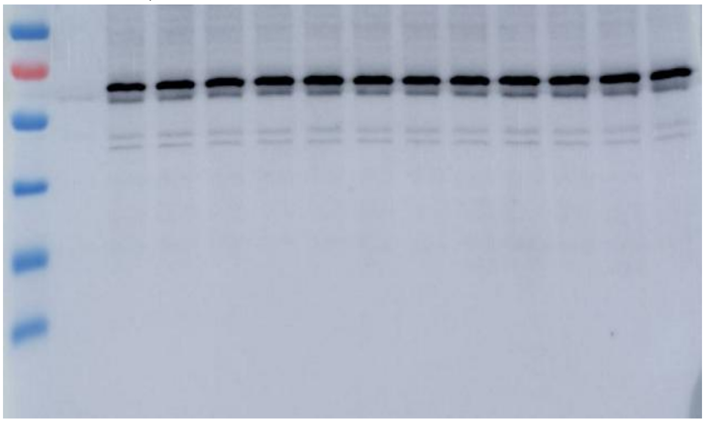

Figure 2C:
IP; HA Ab

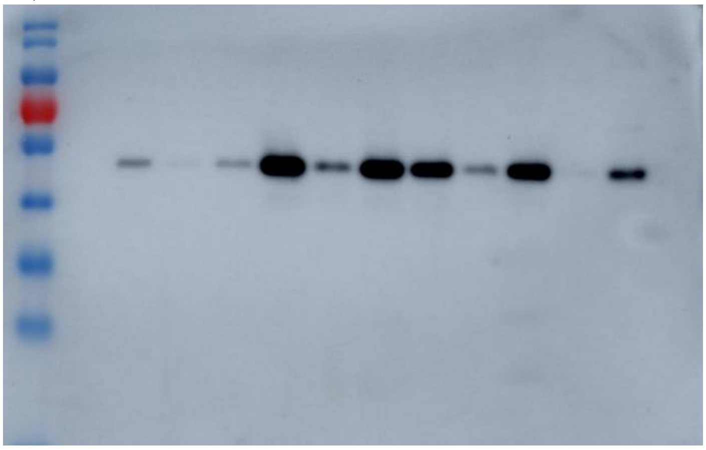

Direct WB; Flag Ab

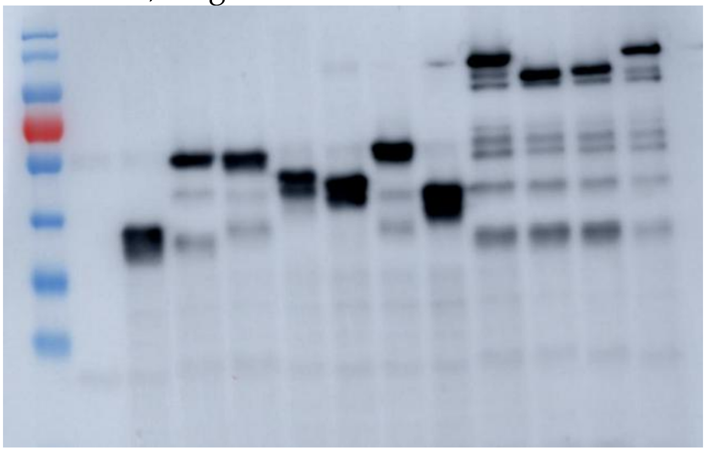

Direct WB; HA Ab

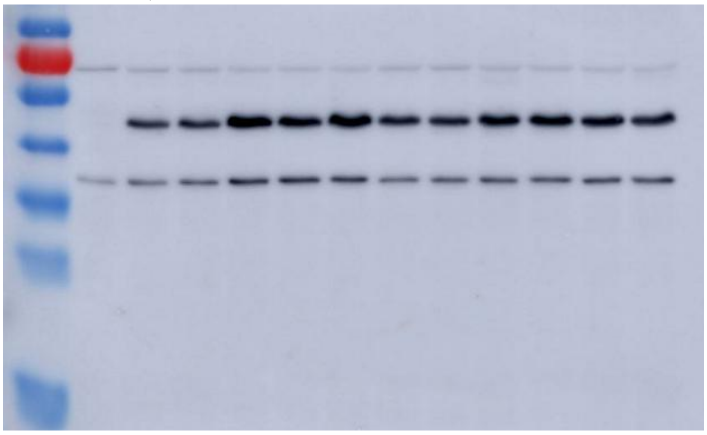

Figure 2E:
IP; HA Ab

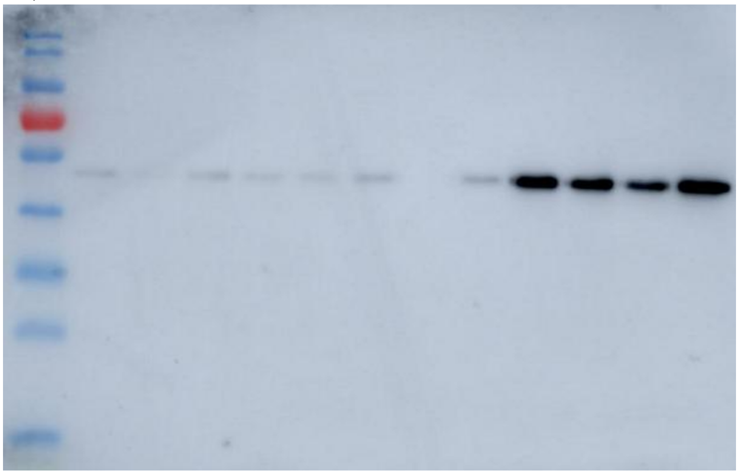

IP; mCherry Ab

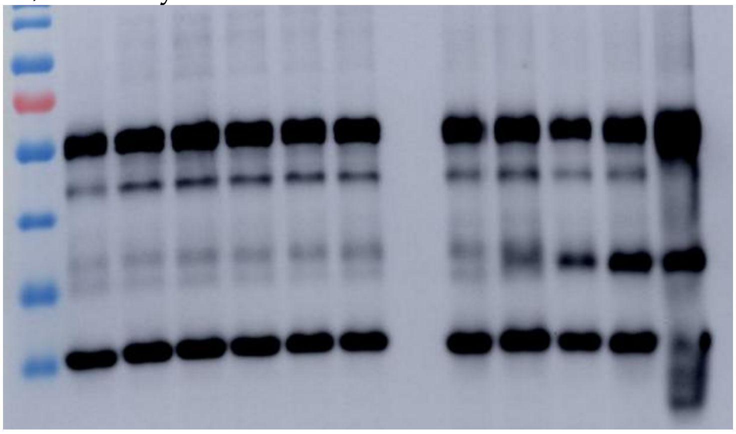

Direct WB; Flag Ab

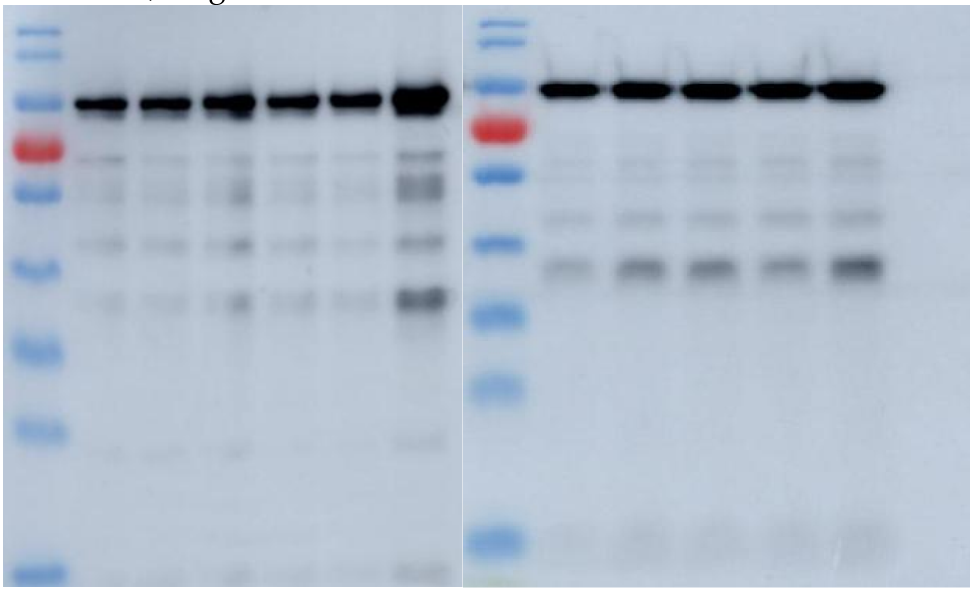

Direct WB; HA Ab

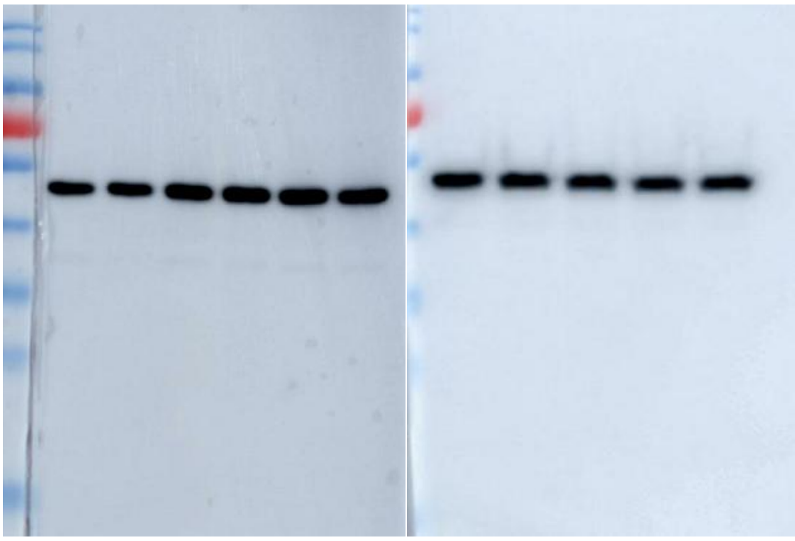

Direct WB; mCherry Ab

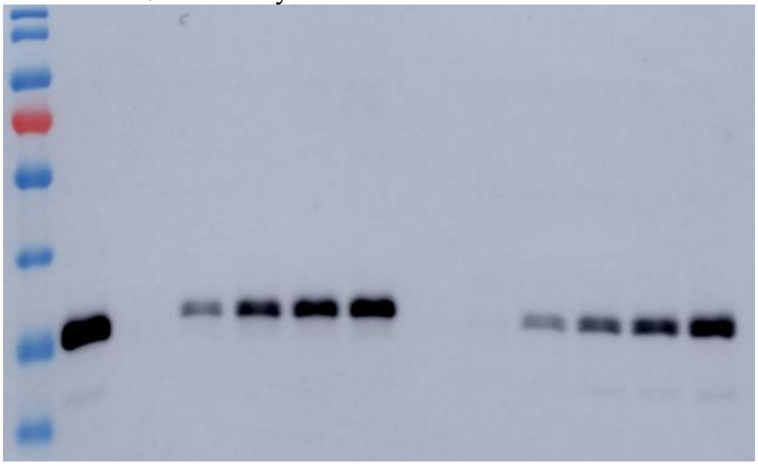

Figure 3B:

IP; HA Ab

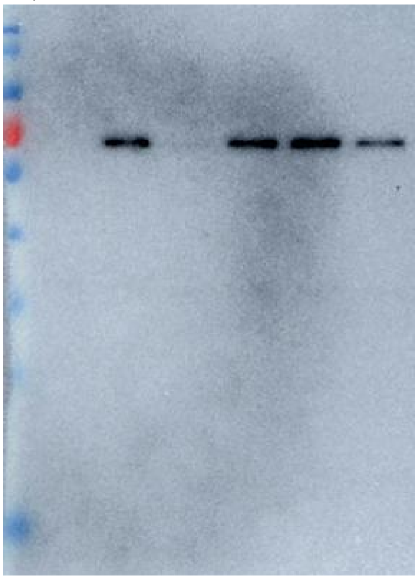

Direct WB; Flag Ab

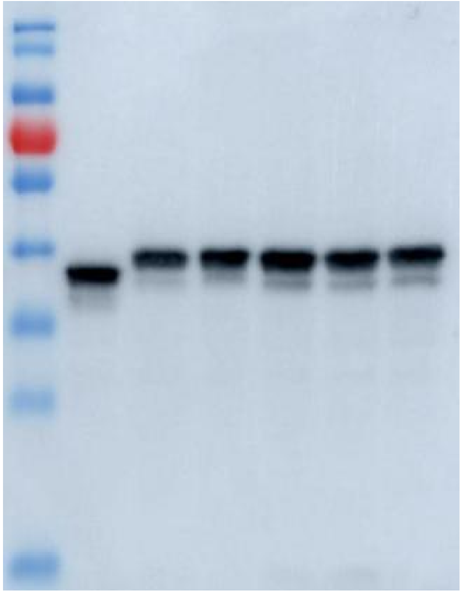

Direct WB; HA Ab

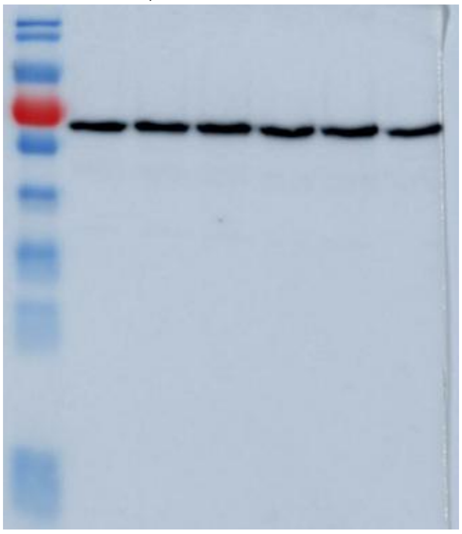

Figure 3C:
IP; HA Ab

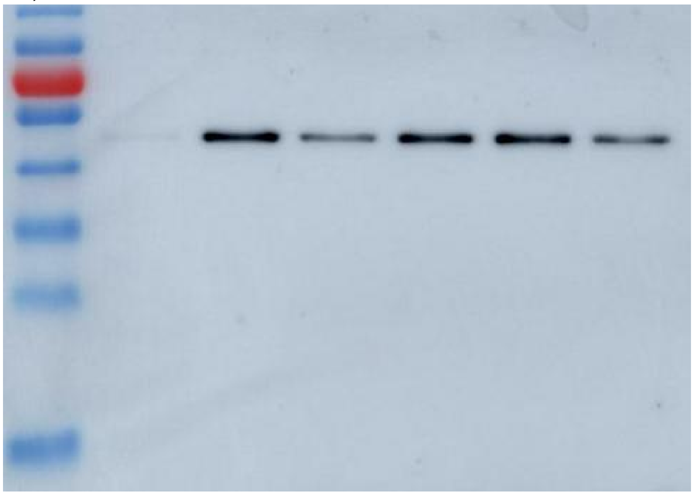

IP; mCherry Ab

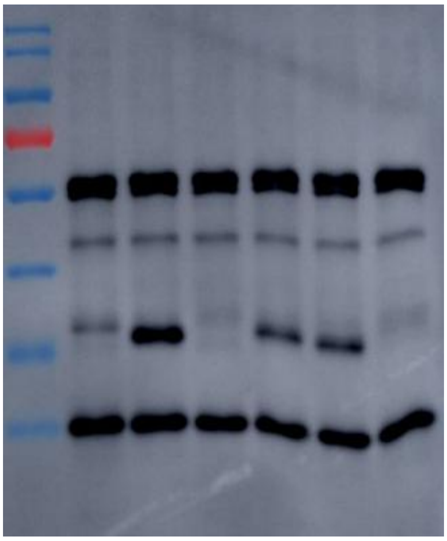

Direct WB; Flag Ab

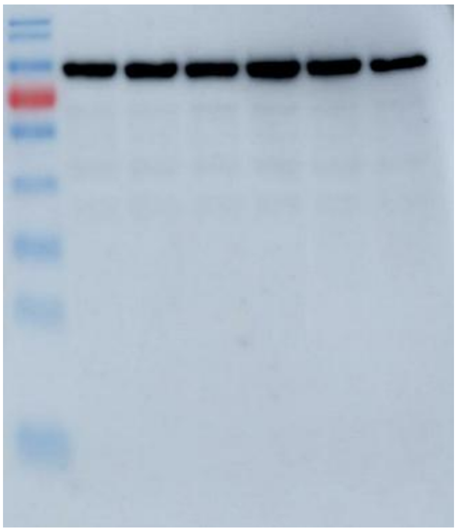

Direct WB; HA Ab

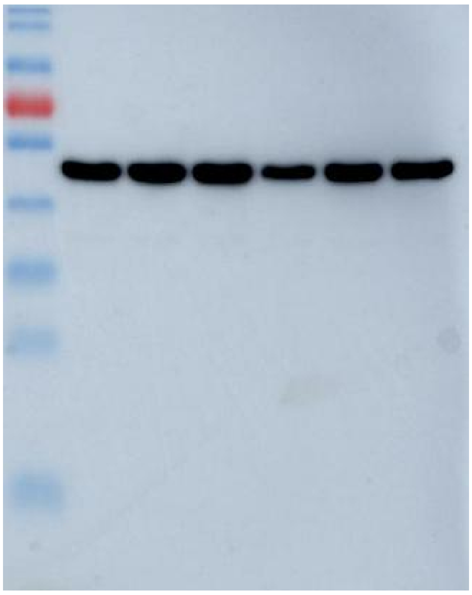

Direct WB; mCherry Ab

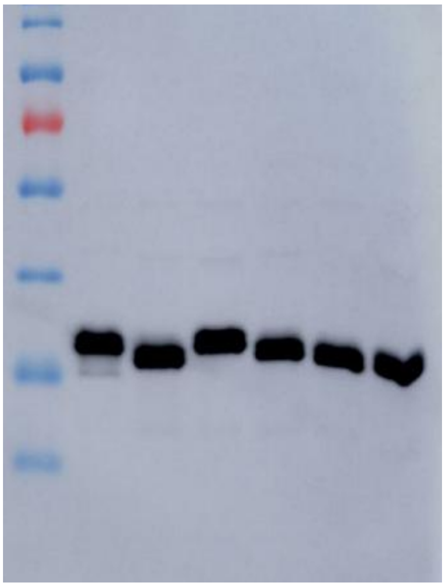

Figure 3D:
IP; HA Ab

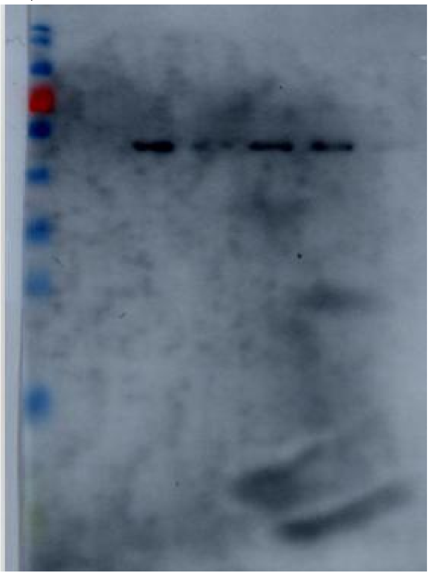

Direct WB; Flag Ab

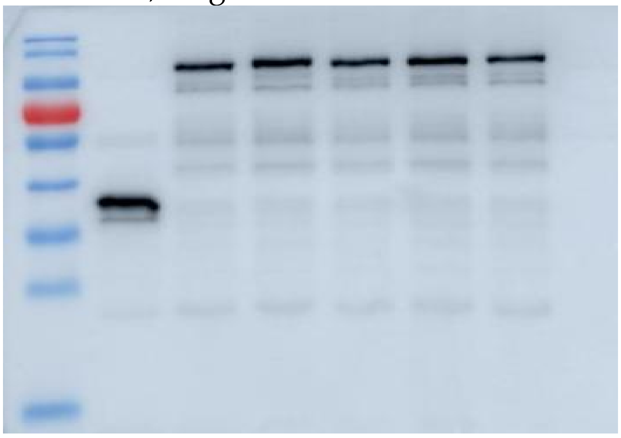

Direct WB; HA Ab

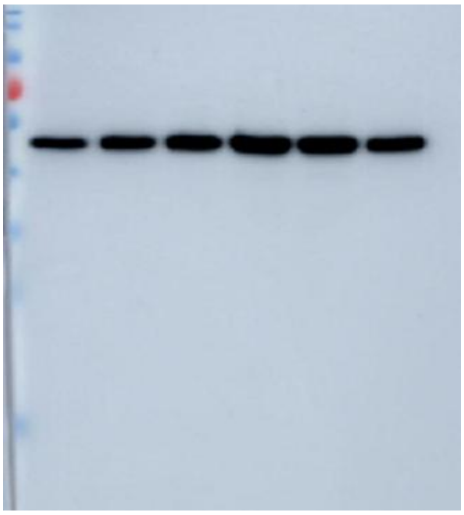

Figure 3F:
Native gel; Flag Ab

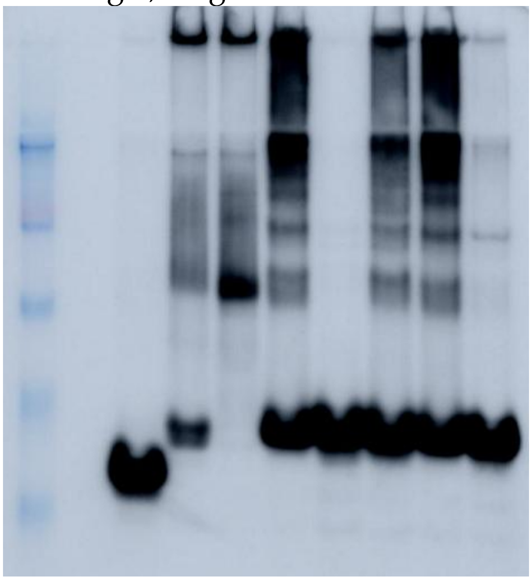

Denatured gel; HA Ab

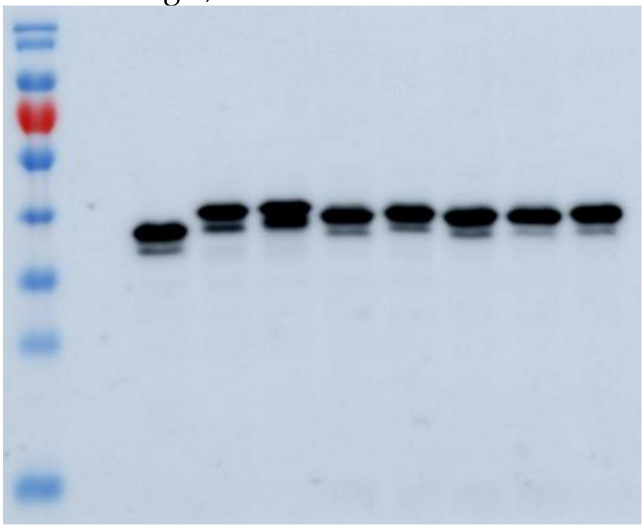

Figure 4B:

Doxycycline (Dox) induced knock-down

YY1 Ab

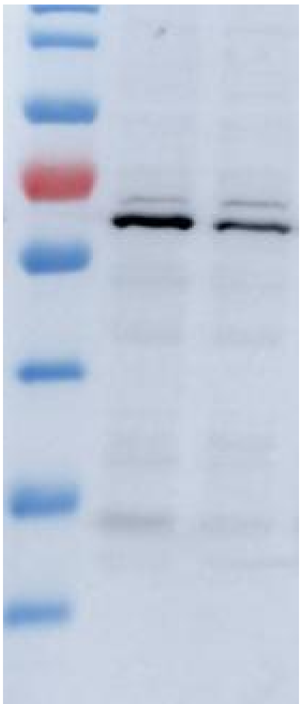

GAPDH Ab

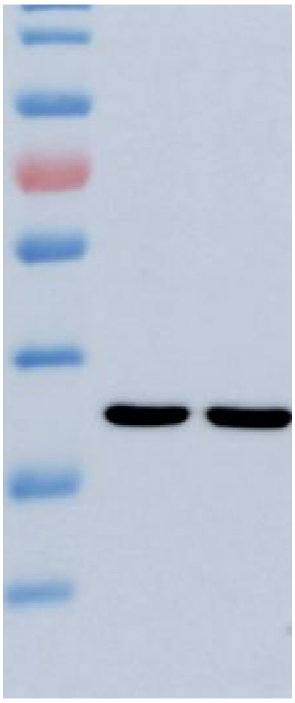

Flag Ab

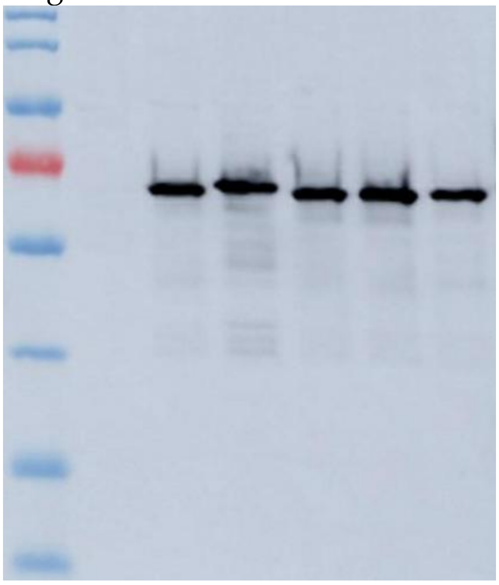

GAPDH Ab

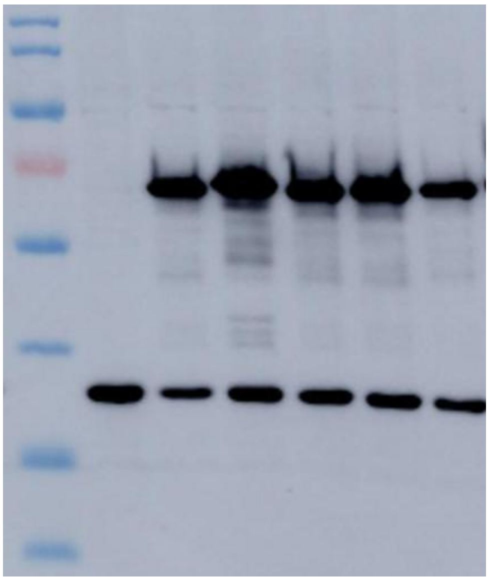

Figure 4D:

Doxycycline (Dox) induced knock-down

YY1 Ab

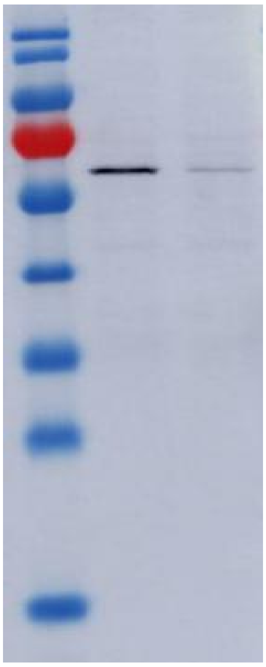

GAPDH Ab

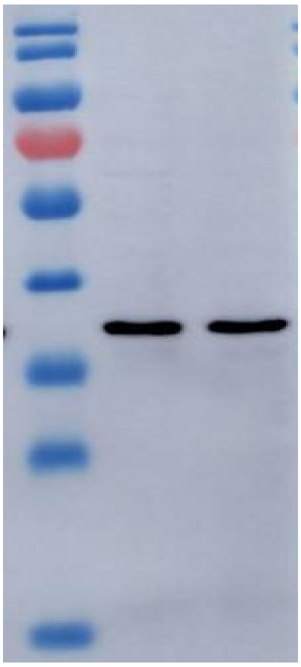

Flag Ab

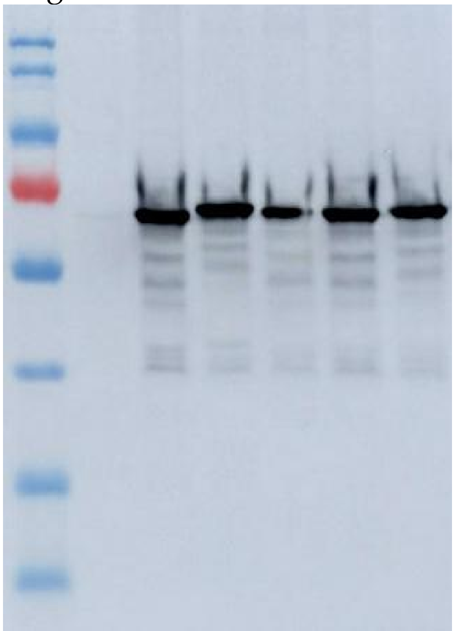

GAPDH Ab

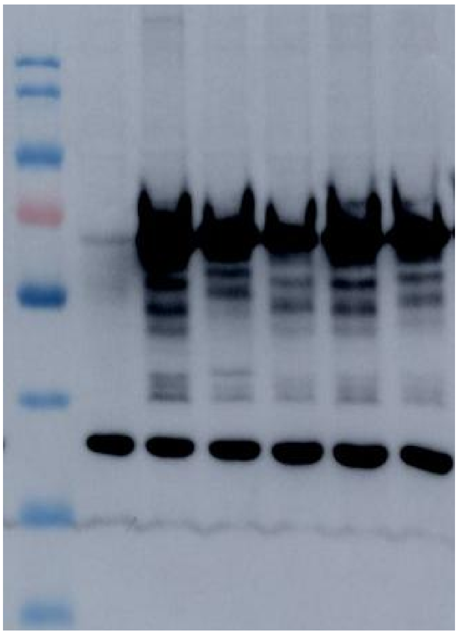

Figure 5A:

His Ab

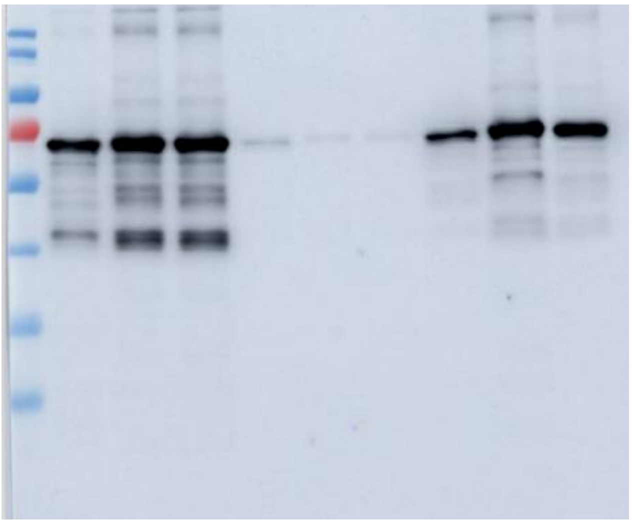

Figure 5B:
IP; EZH2 Ab

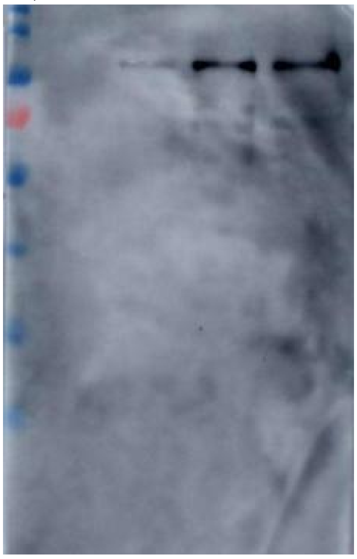

Direct WB; Flag Ab

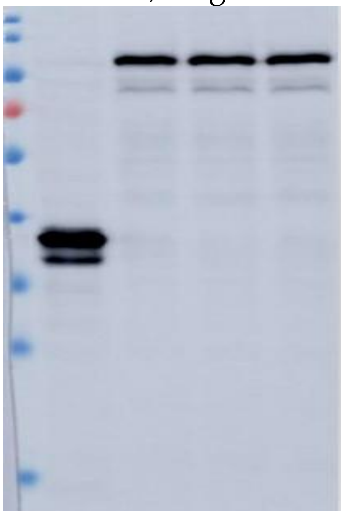

Direct WB; EZH2 Ab

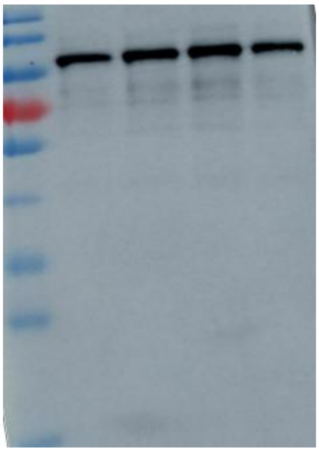

Figure 5C:
IP; HA Ab

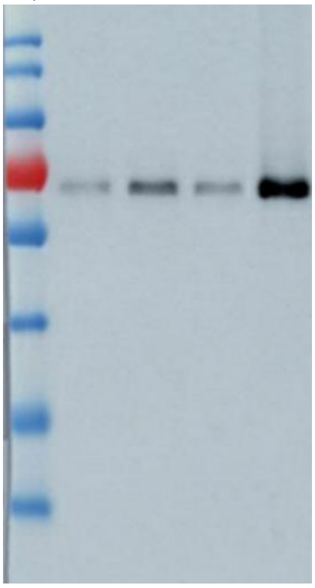

Direct WB; Flag Ab

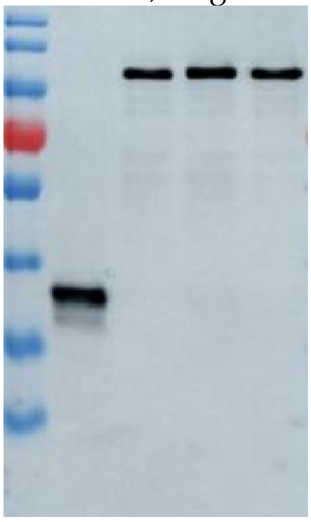

Direct WB; HA Ab

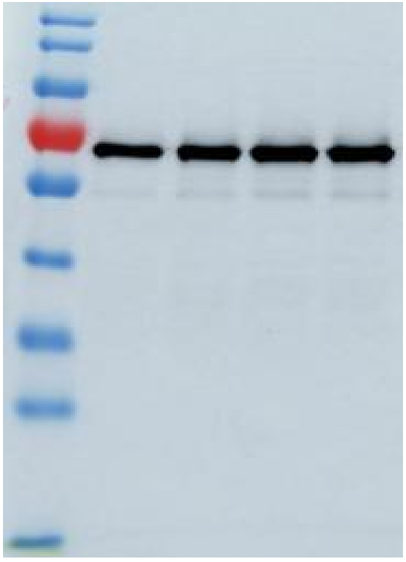

Figure 5D:
IP; HA Ab

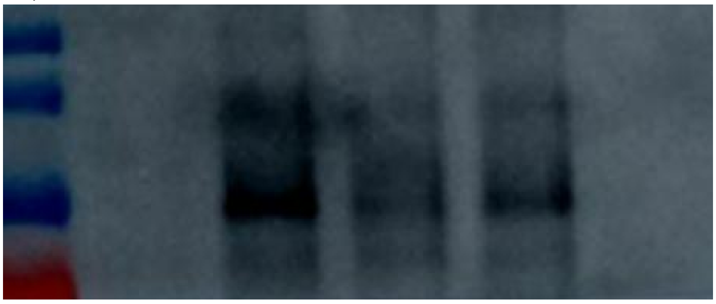

Direct WB; Flag Ab

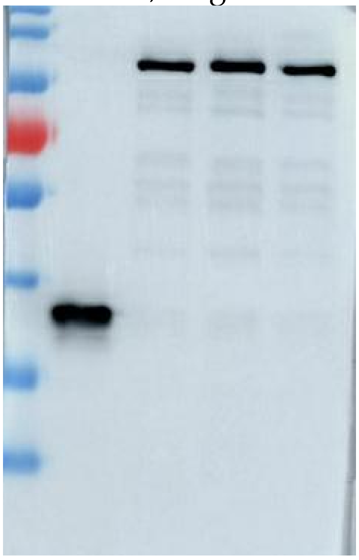

Direct WB; HA Ab

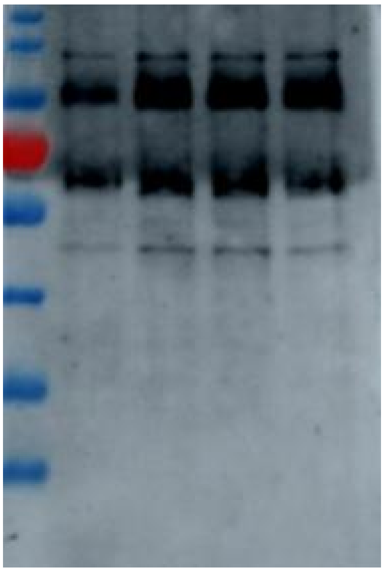

Supplement: Supplementary file 1 [file cancers-14-01611-s001.zip › cancers-1579895-supplementary.pdf]
